# A beacon in the dark: COVID-19 course in CVID patients from two European countries: Different approaches, similar outcomes

**DOI:** 10.3389/fimmu.2023.1093385

**Published:** 2023-02-08

**Authors:** Cinzia Milito, Davide Firinu, Patrick Bez, Annalisa Villa, Alessandra Punziano, Gianluca Lagnese, Giulia Costanzo, Leanne P. M. van Leeuwen, Beatrice Piazza, Carla Maria Deiana, Giancarlo d’Ippolito, Stefano Renato Del Giacco, Marcello Rattazzi, Giuseppe Spadaro, Isabella Quinti, Riccardo Scarpa, Virgil A. S. H. Dalm, Francesco Cinetto

**Affiliations:** ^1^ Department of Molecular Medicine, Sapienza University of Rome, Rome, Italy; ^2^ Department of Medical Sciences and Public Health, University of Cagliari, Cagliari, Italy; ^3^ Rare Diseases Referral Center, Internal Medicine 1, Ca’ Foncello Hospital, AULSS2 Marca Trevigiana, Department of Medicine (DIMED), University of Padova, Padova, Italy; ^4^ Department of Translational Medical Sciences, University of Naples Federico II, Naples, Italy; ^5^ Department of Viroscience, Travel Clinic, Erasmus University Medical Center Rotterdam, Rotterdam, Netherlands; ^6^ Department of Internal Medicine, Division of Allergy and Clinical Immunology, Department of Immunology, Erasmus University Medical Center Rotterdam, Rotterdam, Netherlands

**Keywords:** Common Variable Immunodeficiency (CVID), COVID-19, SARS-CoV-2, precision medicine, outpatient, risk factors

## Abstract

**Background:**

CVID patients present an increased risk of prolonged SARS-CoV-2 infection and re-infection and a higher COVID-19-related morbidity and mortality compared to the general population. Since 2021, different therapeutic and prophylactic strategies have been employed in vulnerable groups (vaccination, SARS-CoV-2 monoclonal antibodies and antivirals). The impact of treatments over the last 2 years has not been explored in international studies considering the emergence of viral variants and different management between countries.

**Methods:**

A multicenter retrospective/prospective real-life study comparing the prevalence and outcomes of SARS-CoV-2 infection between a CVID cohort from four Italian Centers (IT-C) and one cohort from the Netherlands (NL-C), recruiting 773 patients.

**Results:**

329 of 773 CVID patients were found positive for SARS-CoV-2 infection between March 1^st^, 2020 and September 1^st^ 2022. The proportion of CVID patients infected was comparable in both national sub-cohorts. During all waves, chronic lung disease, “complicated” phenotype, chronic immunosuppressive treatment and cardiovascular comorbidities impacted on hospitalization, whereas risk factors for mortality were older age, chronic lung disease, and bacterial superinfections. IT-C patients were significantly more often treated, both with antivirals and mAbs, than NL-C patients. Outpatient treatment, available only in Italy, started from the Delta wave. Despite this, no significant difference was found for COVID-19 severity between the two cohorts. However, pooling together specific SARS-CoV-2 outpatient treatments (mAbs and antivirals), we found a significant effect on the risk of hospitalization starting from Delta wave. Vaccination with ≥ 3 doses shortened RT-PCR positivity, with an additional effect only in patients receiving antivirals.

**Conclusions:**

The two sub-cohorts had similar COVID-19 outcomes despite different treatment approaches. This points out that specific treatment should now be reserved for selected subgroups of CVID patients, based on pre-existing conditions.

## Introduction

From February 2020 to September 2022, more than 250 million people contracted SARS-CoV-2 infection and over 2 million people died from COVID-19 disease in the European Regions (1). The course of disease ranges from asymptomatic/mild to a life-threatening condition (2). In the general population, risk factors for severe COVID-19 disease include older age (>65 years), obesity, male sex, active cancer, arterial hypertension, cardiovascular and respiratory tract diseases (3–6).

Inborn Errors of Immunity (IEIs) are a group of heterogeneous disorders characterized by an impaired host defense, resulting in an increased susceptibility to infections, autoinflammatory complications, autoimmunity and cancer. The constant increase in life expectancy of IEIs patients in the last decades has led clinicians to face new challenges in IEI management, including those linked to COVID-19. Since the beginning of the pandemic, IEIs patients have been considered a potential high-risk group for severe COVID-19. Indeed, an increased risk of hospitalization and mortality associated with COVID-19 in IEIs patients was reported in comparison to the general population (7–9). Within IEIs, Primary Antibody Deficiencies (PADs) are the most prevalent group (approximately 60-70%). Common Variable Immune Deficiency (CVID) is the most frequent symptomatic PAD in adults (10). Over the past years variable clinical presentations of COVID-19 have been reported in CVID patients, ranging from asymptomatic/mild to severe disease or death (7, 11, 12). Differently from the general population, CVID patients presented a lower median age at death and specific predisposing factors to severe COVID-19, including Granulomatous Lymphocytic Interstitial Lung Disease (GLILD) with potential need for immunosuppressive treatment, End-Stage Lung Disease (ESLD) and bronchiectasis (13, 14). In addition, lymphopenia, lower B cell counts, lack of IgG replacement therapy (IgRT) have been reported as factors associated with worse outcome of SARS-CoV-2 infection in patients with CVID (7–9).

In comparison to the general population, CVID patients also present an increased risk of prolonged SARS-CoV-2 infection and re-infection (15). Furthermore, in an Italian cohort, a higher COVID-19-related mortality has been recently demonstrated in patients with severe antibody deficiencies compared to the general population (16).

To prevent SARS-CoV-2 spreading among the general population and vulnerable groups at high risk of severe disease after infection, during the last two years every European country has implemented different rules of social distancing, contact tracing, personal protective equipment usage (PPE), lock-down, self-quarantining and, later on, vaccination.

Immunization is the safest and most effective strategy to achieve a consistent protective immunological response against COVID-19 severity and is strongly recommended also by the European Society for Immunodeficiencies (ESID) for patients with IEIs (17–19). In patients with IEIs, mRNA based COVID-19 vaccines were found to be safe and elicited an antibody and T-cell response in the majority of patients (20–22). Despite the presence of impaired immune responses to many vaccines/antigens in CVID, mRNA vaccines have been associated with the elicitation of T-cell response and atypical or typical B cell pathway activation, particularly after booster doses (20, 23, 24). Of note, lowest response was detected in CVID patients with a complicated clinical phenotype (21). Therapeutic monoclonal antibodies (mAbs) have also been reported as effective and safe for high-risk patients to reduce intensive care admission and hospitalization (25). In addition, different specific antiviral therapies (including remdesivir, nirmatrelvir plus ritonavir, molnupiravir) against SARS-CoV-2 have been developed, also for the outpatient settings (26). The early administration of mAbs and antiviral treatment during the infection has been shown to reduce the risk of severe disease and hospitalization in CVID patients, with antivirals significantly impacting on viral shedding (16, 26, 27).

The purpose of this multicenter retrospective/prospective real-life study is to compare the prevalence of SARS-CoV-2 infection and re-infection and disease outcomes in CVID patients of two different cohorts managed according to different national guidelines: a cohort of patients from four Italian Centers for IEIs (Rome, Naples, Padua and Cagliari) and a Dutch cohort in follow-up at the Primary Immunodeficiency Center in Erasmus University Medical Center Rotterdam. The secondary endpoint is to investigate the impact of different co-morbidities and CVID-related phenotypes, as well as immunization and other therapeutic strategies, on COVID-19 severity and mortality in the last year of the COVID-19 pandemic, in order to improve the management of patients at high risk of developing severe COVID-19.

## Methods

The diagnosis of CVID was established according to the European Society for Immunodeficiencies (ESID) criteria (28). In the study period (March 1, 2020 - September 1, 2022) the spreading of SARS-CoV-2 strains in Europe according to the reports from the National Health Authorities of the two involved countries, was defined as follows: the original Wuhan strain from February to December 2020 (wave 1); the variant B.1.1.7 (Alpha) from January 2021 to mid-July 2021 (wave 2); the variant B.1.617.2 (Delta) from July 2021 to end December 2021 (wave 3) and the B.1.1.529 variant (Omicron) since December 2021 until the end of the study period (wave 4) (24–26). The Italian National Institute of Health report on the SARS-CoV-2 pandemic in Italy and Coronavirus Dashboard from the Government of the Netherlands website, were used to obtain national estimates and data on the general population (29, 30).

SARS-CoV-2 positivity was assessed by approved tests (PCR molecular test and rapid antigen-test with determination of cut off index). Genotype assessment of SARS-Cov-2 variant on nasopharyngeal swabs was not systematically conducted; thus, the infection strain was attributed mostly according to the date of the positive swab. Reinfection was defined as the record of a new positive SARS-CoV-2 test >90 days after the resolution of the first SARS-CoV-2 infection.

According to country regulations, in Italy a SARS-CoV-2 infected subject repeated a test 7-10 days after the first positive swab and then every 3-7 days until a negative test was obtained, in order to stop isolation. The dates of the first positive and first negative SARS-CoV-2 test were recorded in the Italian cohort to evaluate the duration of the RT-PCR positivity.

In the Netherlands, SARS-CoV-2 positive subjects with symptoms had to maintain isolation for 10 days (5 days if asymptomatic), but a negative test was not mandatory to release from quarantine/isolation (31). For this reason, for the Netherlands cohort data about RT-PCR positivity were not available.

We recorded data on sex, age, CVID comorbidities, clinical phenotype, ongoing therapies, COVID-19 disease severity, hospitalization, vaccination status, and SARS-CoV-2 specific treatments. End-stage lung disease was defined as chronic oxygen dependent respiratory failure. Chronic immunosuppressive treatment was defined as receiving steroids and/or DMARDS, and/or biologics for immunosuppressive purposes. According to Chapel’s criteria, the CVID cohort was divided into two different clinical phenotypes: “infection only” and “complicated’’ (32). All patients enrolled in the study were on IgRT. COVID-19 severity was defined according to WHO classification (WHO Working Group) (33). All vaccinated patients received a mRNA vaccine, namely BNT162b2 in Italy and mRNA-1273 in the Netherlands. We defined as “home treatment” the outpatient administration of antivirals (remdesivir, nimatrelvir/ritonavir, molnupinavir) and/or mAbs (bamlanivimab, bamlanivimab/etesivimab, casirivimab/imdevimab; sotrovimab after the spread of Omicron), according to the availability and indication for outpatient administration (27, 34). These treatments were available for early outpatient use in Italy, but not in the Netherlands, for all CVID patients infected by SARS-CoV-2 with at least mild symptoms. As far as mAbs are concerned, specific anti-SARS-CoV-2 antibodies assessment was not required before outpatient treatment administration in patients with primary antibody deficiency. The study was approved by the Local Ethical Authorities and was performed in accordance with Good Clinical Practice guidelines, the International Conference on Harmonization guidelines, and the most recent version of the Declaration of Helsinki.

### Statistical analysis

Patients' characteristics were summarized using medians, standard deviations, interquartile ranges, and percentages as appropriate. Chi-squared tests of independence and Fisher’s exact tests were used for categorical data. Mann-Whitney U and Kruskal-Wallis tests were used for unpaired continuous data. Binomial logistic regression models were fitted to calculate odds ratios (OR) with 95% confidence intervals (CI) for the need of hospitalization and the presence of severe disease in association with mAbs or antiviral administration. Multivariable logistic regression analysis was then performed, to confirm the findings, taking into consideration age, sex and co-morbidities. Statistical significance was considered as a two-tailed p<0.05. All the analyses were performed using IBM SPSS statistics 28.0.

## Results

### Patients’ characteristics

From 1st March 2020 to 1st September 2022, a total of 773 CVID patients were recruited: a cohort of 497 in follow-up at four Italian Referral Centers for IEIs (Rome, Naples, Padua, and Cagliari) and a cohort of 276 in follow-up at Erasmus University Medical Center Rotterdam (the Netherlands). During the study period, 329/773 (42.5%) of the whole cohort got infected by SARS-CoV-2. The prevalence of infection was similar in the IT-C (Italian Cohort) and NL-C (the Netherlands Cohort) cohort: 218/497 (43.9%) infected patients from the IT-C and 111/276 patients (40.2%) from the NL-C were recorded (p=0.362).

**Figure 1:**
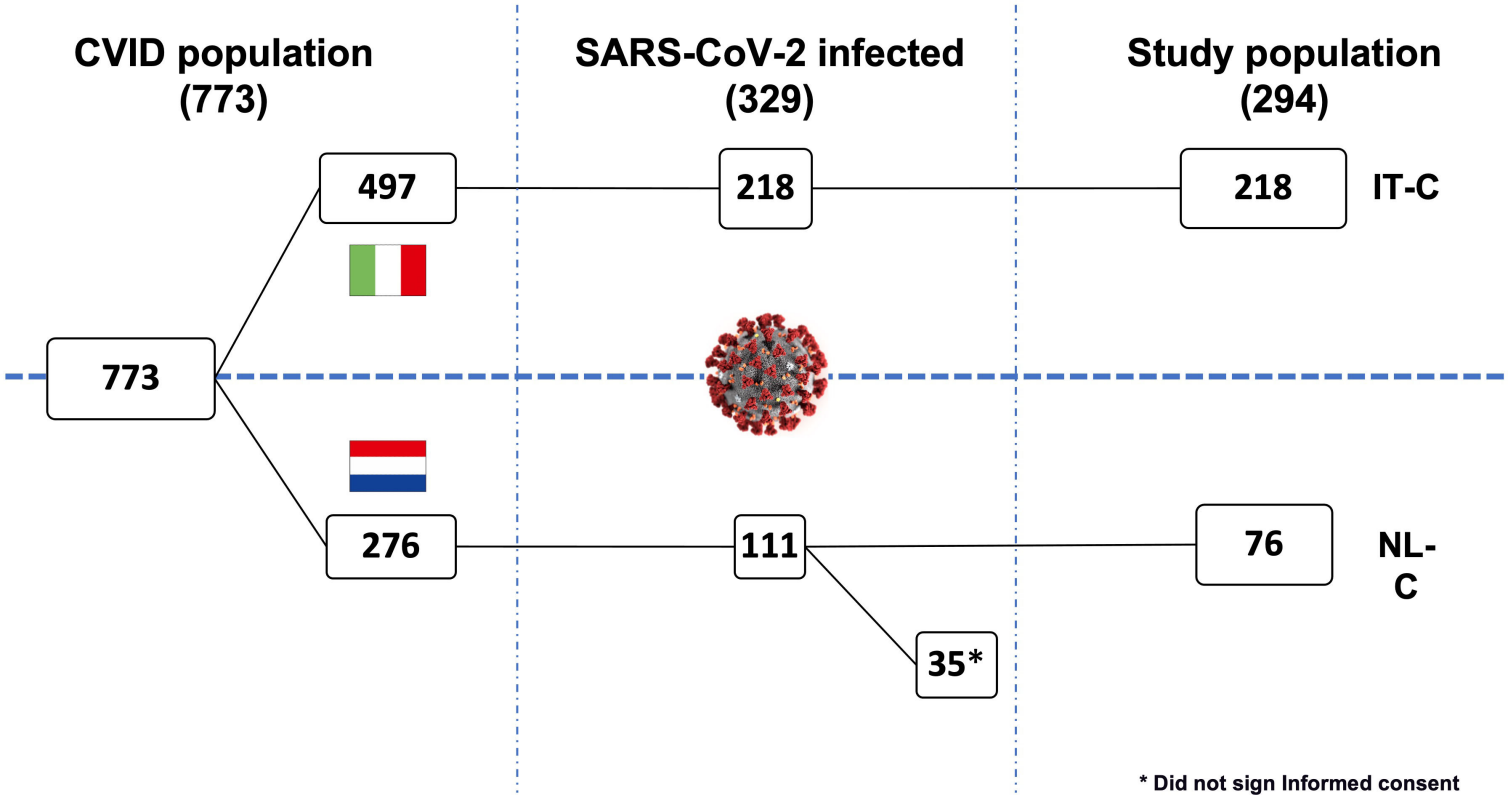
Study design.

Data regarding the CVID cohort and general population in Italy and the Netherlands are recapitulated in **Supplementary Table 1**. Of note, while infection rate was in line with that of the general population, the overall mortality rate was 2-3 times higher. While in the IT-C 100% of infected patients gave informed consent to collect further data for this specific study, in the NL-C 76 CVID patients (68.5%) provided consent. Thus, 294 patients (89.4% of the infected patients) were included in the final analysis (**Figure 1**).

Considering the progressive spread of SARS-CoV-2 variants of concern (VOC)s, 22.1% of the whole cohort got infected during the spreading of Wuhan and Alpha strains. During the Delta wave 8.8% of the patients got infected, and 69.4% during the Omicron wave. Comparing our sub-cohorts, IT-C presented a lower percentage of SARS-CoV-2 cases than NL-C in the Delta wave (6.0% *vs* 17.1%; p=0.003), whereas during Omicron wave IT-C presented significantly more cases than NL-C (72.9% *vs* 59.2%; p=0.025). Demographics and CVID-related information are recapitulated in **Table 1**. A known CVID-associated genetic variant was found in 4.4% of the enrolled patients, TACI being the most commonly involved gene. We did not include patients with known monogenic forms of CVID in the cohort.

**Table 1:** Demographics and CVID-related information in the whole cohort and in the IT-C and NL-C sub-cohorts.

ENROLLED PATIENTS (%)	Whole cohort	IT-C	NL-C	p
	294 (89.36)	218 (100)	76 (68.46)	0.362
Median Age (IQR)	50 (37-60)	50.5 (40-61.25)	47 (28-58)	**0.018**
Sex F (%)	165 (56.1)	127 (58.3)	39 (51.31)	0.190
Wuhan+Alpha (%)	65 (22.1)	47 (21.6)	18 (23.7)	0.701
Delta (%)	26 (8.8)	13 (6)	13 (17.1)	**0.003**
Omicron (%)	204 (69.4)	159 (72.9)	45 (59.2)	**0.025**
Chronic Lung disease (%)	124 (42.2)	88 (40.3)	36 (47.3)	0.287
- Bronchiectasis (%)	111 (37.7)	85 (38.9)	26 (34.2)	0.494
- GLILD (%)	46 (15.6)	29 (13.30)	17 (22.37)	0.068
- ESLD (%)	7 (2.4)	5 (2.29)	2 (2.63)	1.000
Complicated phenotype (%)	112 (38.1)	91 (41.3)	21 (27.6)	**0.039**
AI Cytopenia (%)	59 (20.0)	48 (22.0)	11 (14.5)	0.184
Chronic Immunosuppressive treatment	39 (13.2)	28 (9.5)	11 (14.5)	0.729
Lymphocytes (cells/mmc)	1460 (1115-2035)	1460 (1157-2012.5	1405 (965-2357.50)	0.662
IgG-TL in mg/dl (IQR)	743 (572-899)	720 (569-865)	1010 (775-1170)	**<0.001**

The bold values refers to statistical significant data when the corresponding p value is < 0.05.

The median age of SARS-CoV-2 positive patients was 50 years (range 37-60), with 19.4% of patients (57/294) older than 65 years. NL-C patients were significantly younger than the ones of IT-C [50.5 (40–61) *vs* 47 (28–58); p=0.0176]. In the whole cohort, 56.1% (165/294) of infected patients were females, with no difference between the national sub-cohorts.

Concerning pre-existent lung damage, 42.2% of SARS-CoV-2 positive patients presented one or more lung comorbidities: 37.7% had bronchiectasis, 15.6% GLILD and 2.4% ESLD. A “complicated” clinical phenotype was identified in 38.1% of infected patients, with a higher prevalence in the IT-C (41.3% *vs* 27.6%, p=0.039). Autoimmune cytopenias were registered in 59 patients (20.0%); in particular, 53 (18.0%) had or previously presented immune thrombocytopenia (ITP) and 13 (4.4%) autoimmune hemolytic anemia (AIHA). The prevalence of the above-mentioned CVID-related comorbidities was similar between the 2 sub-cohorts, with a higher but not statistically different prevalence of GLILD in the NL-C (13.3% *vs* 22.4%, p=0.068). Of note, the prevalence of ongoing chronic immunosuppressive treatment was also similar between the two cohorts, being mostly (>80%) represented in patients with a complicated phenotype.

Regarding laboratory features, in the whole population the median value of lymphocytes before COVID-19 was 1460 cells/mm^3^ (range 1115-2035) and the median IgG trough level (IgG-TL) was 743 mg/dL (range 572-899) with a significantly higher IgG-TL in the NL-C when compared with the IT-C (720 mg/dL *vs* 1010 mg/dL p<0.001) (**Table 1**).

The prevalence of cardiovascular comorbidities, well-known risk factors for a severe COVID-19 course in the general population, was not different between the 2 sub-cohorts (**Supplementary Table 2**).

### Course of COVID-19

#### Disease severity

Data on disease severity, vaccination status at infection and different adopted treatments are recapitulated in **Table 2**. A total of 256 patients (87.1%) had an asymptomatic or mild disease course, 28 (9.5%) had a moderate and 9 (3.1%) had a severe course, with no significant difference between the two cohorts. The median duration of SARS-CoV-2 infection, available only for the IT-C cohort, was 15.5 days (range 10-23.25).

**Table 2:** COVID-19 course and treatments.

ENROLLED PATIENTS (%)	Whole cohort (N=294)	IT-C (N=218)	NL-C (N=76)	p value
	294 (89.4)	218 (100)	76 (68.5)	
SARS-CoV-2 SEVERITY				
Asympt/mild (%)	256 (87.1)	192 (88.07)	64 (84.2)	0.387
Moderate (%)	28 (9.5)	19 (8.7)	9 (11.84)	0.496
Severe (%)	9 (3.1)	6 (2.8)	3 (3.95)	0.700
Hospitalized (%)	37 (12.6)	25 (11.5)	12 (15.78)	0.328
ICU (%)	7 (2.4)	7 (3.2)	0	0.196
Died (%)	5 (1.7)	4 (1.8)	1 (1.3)	1.000
Bacterial superinfections (%)	21 (7.1)	16 (7.3)	5 (6.6)	1.000
PTE (%)	1 (0.34)	1 (0.35)	0	1.000
Acute CV Events (%)	1 (0.34)	1 (0.35)	0	1.000
SARS-CoV-2 DURATION				
Median days (IQR)	15.5 (10-23.25)	15.5 (10-23.25)	N/A	
Hospitalization days (IQR)	11 (5-28)	14 (7-29)	5.5 (2.5-10.25)	**0.003**
SARS-CoV-2 TREATMENT				
Hyperimmune Plasma (%)	4 (1.4)	3 (1.4)	1 (1.3)	1.000
Antiviral (%)	72 (24.5)	70 (32.1)	2 (2.6)	**<0.001**
Antiviral home (%)	57 (19.4)	57 (26.1)	0	**<0.001**
mAbs (%)	80 (27.2)	74 (33.9)	7 (9.2)	**<0.001**
mAbs home (%)	67 (22.8)	67 (30.7)	0	**<0.001**
mAbs hosp (%)	17 (5.8)	10 (4.6)	7 (9.2)	0.162
Home treatment (%)	122 (41.5)	122 (55.9)	0	**<0.001**
IV steroids (%)	23 (7.8)	16 (7.3)	7 (9.2)	0.632
Tocilizumab (%)	3 (1.0)	2 (0.9)	1 (1.3)	1.000
Azithromycin (%)	25 (8.5)	25 (11.5)	0	**<0.001**
Other ABT (%)	18 (6.1)	8 (3.7)	10 (13.1)	**0.009**
IMMUNIZATION status at infection				
Not vaccinated (%)	79 (26.9)	59 (27.0)	20 (26.3)	0.899
at least 2 doses (%)	216 (73.5)	160 (73.4)	56 (73.7)	0.961
at least 3 doses (%)	193 (65.6)	146 (66.9)	47 (61.8)	0.483
4 doses (%)	42 (14.3)	26 (11.9)	16 (21.1)	0.058

The bold values refers to statistical significant data when the corresponding p value is < 0.05.

Thirty-seven out of 294 patients (12.6%) were hospitalized, 11.5% in the IT-C and 15.8% in the NL-C respectively, with no significant difference. The median days of hospitalization were 11 (range 5-28), with a significantly longer hospital stay in IT-C than the NL-C [14 days (7–29) vs. 5.5 days (2.5-10.3); p=0.003]. Intensive care unit (ICU) admission was necessary for 7 Italian patients (median age at ICU admission 52.3 years; 2.4% of the whole population). A total of 5 patients died (median age 59 years) after a median of 16 hospital days; 4 patients died in the IT-C (1.8% of enrolled population) and 1 patient in NL-C (1.3% of enrolled population). A detailed description of dead patients is reported in **Supplementary Table 3**. Complete data about severity, treatment and vaccination during different SARS-CoV-2 waves with the comparison between Italy and the Netherlands, confirming a similar outcome, are presented in **Supplementary Tables 4A–D**.

Moving to SARS-CoV-2 specific treatments, and due to the different country policies, IT-C patients were significantly more frequently treated than NL-C patients, both with antiviral therapy (70 patients *vs* 0; p<0.001) and mAbs (74 *vs* 7; p<0.001) (**Table 2**). In Italy, home/outpatient administration of mAbs and antivirals became available for immunocompromised patients during the Delta wave and widely used during the Omicron wave. Thus, 57 patients were treated with antiviral drugs and 67 patients with mAbs at home (**Table 2**). Noteworthy, we observed that patients with “complicated” phenotype received significantly more often the home/outpatient treatment (50.9% *vs* 35.7%; p=0.010).

#### Efficacy of SARS-CoV-2 specific treatments and impact of vaccination

Considering the period of Delta and Omicron strains dominance, the home administration of SARS-CoV-2 specific treatments globally reduced the hospitalization rates both in patients treated with mAbs (Fisher p=0.029) and antiviral therapy (Fisher p=0.049) (**Table 3**). This effect was not confirmed by univariate logistic regression analysis (**Supplementary Table 5**). When considering the Omicron wave alone, no significant effect of either mAbs or antiviral treatment was found anymore (Fisher p=0.120 and p=0.116, respectively) (**Table 3** and **Supplementary Table 6**). However, when pooling together mAbs and antiviral home treatment, a significant effect on the risk of hospitalization was found both for Delta plus Omicron and for Omicron alone waves (Fisher p<0.001 and p=0.007, respectively; **Table 3**). This effect was confirmed by univariate logistic regression analysis, even when corrected for sex and age (**Supplementary Tables 5**, **6**). Of note, when considering the Delta plus Omicron and Omicron alone waves, in the IT-C home treatment was used in a significantly higher proportion of patients with complicated phenotype (61.4% vs 43.3%, p=0.008 and 68.5% vs 45.4%, p=0.002, respectively).

**Table 3:** Chi square/Fisher exact test for hospitalization considering the period of Delta and Omicron wave together and of Omicron wave alone.

	Delta and Omicron period (p value)	Omicron period (p value)
**Complicated phenotype** **vs uncomplicated**	**0.007**	**0.002**
**Immunosuppressive treatment *vs* untreated**	**<0.001**	**<0.001**
**mAbs home *vs* no mAbs home**	**0.029**	0.120
**Antiviral home *vs* no antiviral home**	**0.049**	0.116
**Any Home treatment *vs* no home treatment**	**<0.001**	**0.007**
**Vaccinated** **(at least 2 doses) *vs* less than 2 doses**	1.000	1.000
**3 doses *vs* less than 3 doses**	0.203	0.368

The bold values refers to statistical significant data when the corresponding p value is < 0.05.

Considering the whole cohort, at the time of SARS-CoV-2 infection, 26.9% of the patients were unvaccinated, 73.5% had received at least 2 doses of vaccine, 65.6% at least 3 doses and 14.3% 4 doses. The vaccination status at infection was similar in the two sub cohorts (**Table 2**). The vaccination status did not show an impact on the risk of hospitalization starting from the Delta wave (Delta + Omicron period) and also during the Omicron wave alone (**Table 3** and **Supplementary Tables 5**, **6**).

#### RT-PCR positivity

Data on RT-PCR positivity were available only for the IT-C. The median time of swab positivity decreased progressively over time, being 22.0 days (16.0-30.5) for the Wuhan-Alpha period, 17.0 days (13.5-20.0) for the Delta period and 14.0 days (9.8-22.0) for the Omicron period. Of note, 16 patients (7.4%) experienced a positivity longer than 40 days. One patient tested positive for 84 days (**Supplementary Table 7**).

When considering patients infected by Delta or Omicron variants, vaccination with 2 doses did not significantly impact duration of positivity (p=0.140). In patients vaccinated with at least 3 doses time to negative testing was shorter when compared to those with less than 3 doses [13.00 days (9.0-21.75) vs 20.0 days (11.0-30.0); p=0.029] and similar results were found comparing patients immunized with 4 doses and those with 3 or less [10 days (7.0-14.8) vs 15 days (10.0-23.0); p=0.001]. Antiviral treatment significantly reduced disease duration in patients vaccinated with at least 3 doses, compared to patients who received no home treatment or mAbs home treatment [11 days (7.0-14.25) vs 15 (11-24.5); p<0.001].

During the spread of Omicron variant, antiviral treatment was still effective in reducing the duration of positive swab tests in patients vaccinated with at least 3 doses [10.0 (7.0-14.5)] both versus mAbs-home-treated [19.0 days (12.0-26.0); p<0.001] and all other patients (mAbs + no treatment) [16.0 days (11.0-25.0); p<0.001] as well as versus untreated [13.0 days (10.0-21.0); p=0.024]. Disease duration was not significantly different between mAbs and no-home-treatment groups (p=0.052). In patients immunized with 4 doses, antiviral home treatment did not significantly shorten the time of swab positivity [11.0 days (7.0-16.0) *vs* 17.0 (11.0-24.0); p=0.103].

#### Complications

During the course of infection only one patient had PTE, one had an acute cardiovascular event, while 7.1% of the infected patients faced a bacterial superinfection, with no difference between the two sub-cohorts (**Table 2**). In the whole cohort hospitalization (p<0.001), chronic lung disease (p=0.005), bronchiectasis (p<0.001), GLILD (p=0.002), ESLD (p<0.001), complicated clinical phenotype (p=0.019), chronic immunosuppressive treatment (p<0.001) and previous cardiovascular events (p=0.011) showed a significant association with superinfections at Fisher exact test (**Supplementary Table 8**). Lower B-cell percentage (2% (1–4); p=0.002) was found in patients who developed superinfections, while IgG-TL and IgA levels were not significantly different. B- cell % impacted on the probability of superinfection (OR 0.769, 95%CI 0.628-0-943; p<0.012), also when adjusting for age and sex (OR 0.762, 95%CI 0.618-0.939; p<0.011).

The duration of SARS-CoV-2 infection also impacted on the probability of superinfection in the whole Italian cohort (OR, 1.108, 95%CI 1.051-1.168; p<0.001), even when adjusting for age and sex (OR 1.107, 95%CI 1.051-1.166; p<0.001). The impact was confirmed during the Omicron period and after adjusting for age, sex and complicated clinical phenotype (OR 1.090, 95%CI 1.034-1.149; p=0.001).

#### Predictive factors of hospitalization and mortality

**Figure 2:**
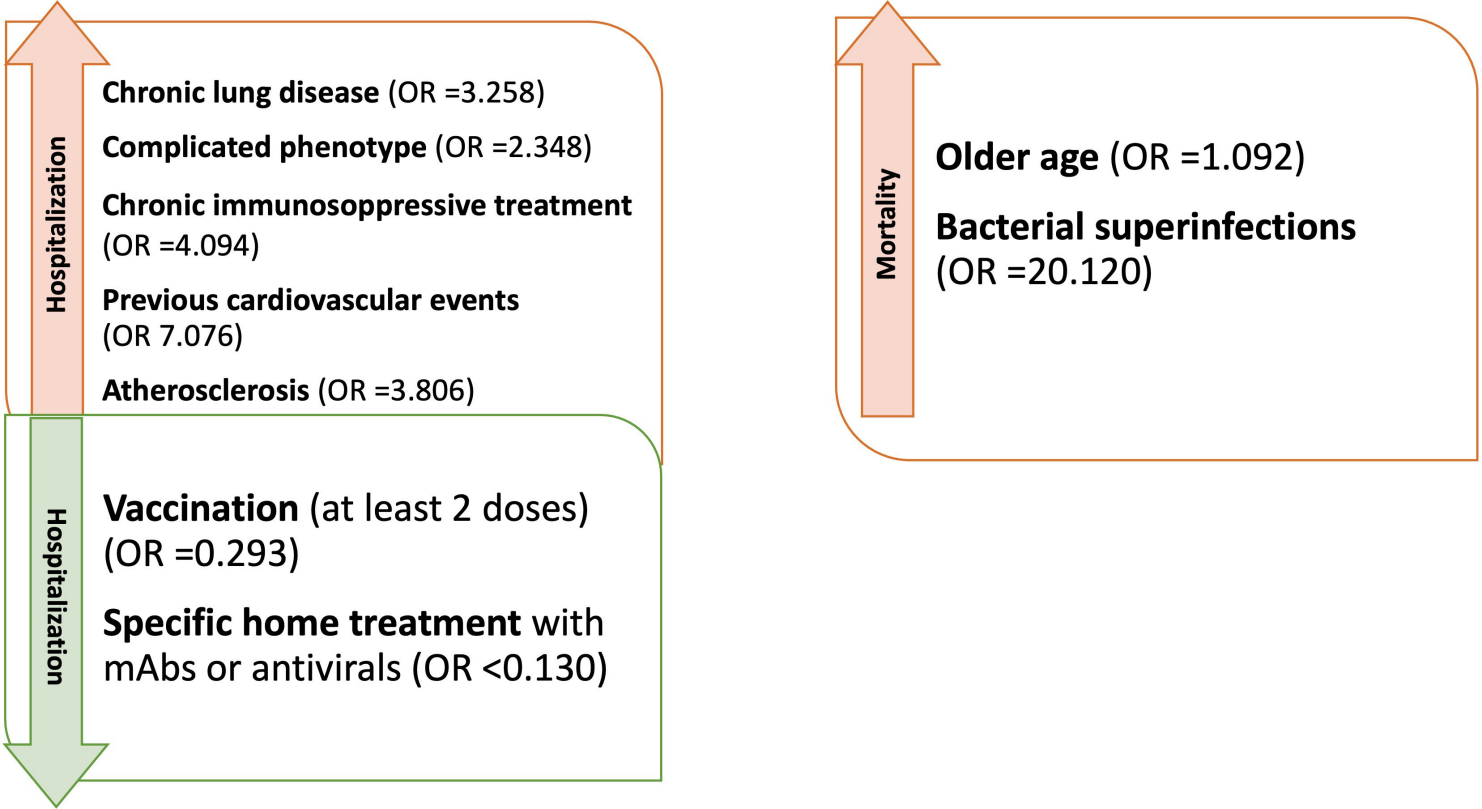
Factors influencing hospitalization and mortality in CVID patients with Sars-CoV-2 during the entire period of observation (OR adjusted for sex and age).

Analyzing the risk factors for hospitalization in the whole population, chronic lung disease (OR 3.325, 95%CI 1.598-6.918); p=0.001), ESLD (OR 49.548, 95%CI 5.774-425.18; p<0.001), GLILD (OR 3.148, 95%CI 1.447-6.848; p=0.004), bronchiectasis (OR 3.155, 95%CI 1.547-6.434; p=0.002) and “complicated” phenotype (OR 2.394, 95%CI 1.190-4.816); p=0.014) and chronic immunosuppressive treatment (OR 4.259, 95%CI 1.915-9.474; p<0.001) impacted on this outcome in the whole study period. Chronic lung disease (OR 4.229, 95%CI 1.873-9.866; p<0.001) and chronic immunosuppressive treatment (OR 3.201, 95%CI 1.298-7.897; p=0.012) retained significance in a multiple regression model, also when adjusted for the other risk factors highlighted by the univariate analysis. Dealing with other comorbidities, atherosclerotic disease (OR 3.862, 95%CI 1.532-9.735; p=0.004), arterial hypertension (OR 2.236, 95%CI 1.079-4.631; p=0.030) and previous cardiovascular events (OR 6.536, 95%CI 2.531-16.876; p<0.001) impacted significantly on hospitalization. All these data, apart from arterial hypertension, were confirmed after adjusting for sex and age (**Table 4** and **Figure 2**). Almost all the aforementioned correlations retained statistical significance also during Delta + Omicron and Omicron waves, with only bronchiectasis losing significance during the Omicron wave (**Supplementary Tables 5**, **6**).

**Table 4:** Univariate analysis: hospitalization as outcome in the whole CVID cohort during the entire period of observation.

Whole cohort	Unadjusted		Adjusted for age and sex	
	p value	OR (IC95%)	p value	OR IC(95%)
**Sex**	0.694	1.151 (0.571-2.320)	0.827	1.083** (0.530-2.213)
**Age**	0.335	1.011 (0.989-1.034)	0.365	1.010* (0.988-1.033)
**Chronic Lung Disease**	**0.001**	3.325 (1.598-6.918)	**0.002**	3.258 (1.550-6.849)
**Bronchiectasis**	**0.002**	3.155 (1.547-6.434)	**0.002**	3.091 (1.501-6.366)
**GLILD**	**0.004**	3.148 (1.447-6.848)	**0.004**	3.110 (1.425-6.788)
**ESLD**	**<0.001**	49.548 (5.774-425.18)	**<0.001**	47.761 (5.534-412.210)
**Complicated phenotype**	**0.014**	2.394 (1.190-4.816)	**0.017**	2.348 (1.165-4.732)
**Chronic Immunosuppressive treatment**	**<0.001**	4.259 (1.915-9.474)	**<0.001**	4.094 (1.815-9.235)
**IgG-TL**	0.137	1.001 (1.000-1.002)	0.087	1.001 (1.000-1.003)
**Age>65**	0.713	1.171 (0.504-2.719)	0.767	1.139* (0.483-2.686)
**Obesity**	0.389	1.522 (0.585-3.957)	0.439	1.461 (0.559-3.814)
**Arterial hypertension**	**0.030**	2.236 (1.079-4.631)	0.057	2.295 (0.975-5.402)
**Diabetes**	0.808	1.172 (0.328-4.188)	0.962	1.032 (0.281-3.789)
**Previous CV events**	**<0.001**	6.536 (2.531-16.876)	**<0.001**	7.076 (2.525-19.835)
**Atherosclerosis**	**0.004**	3.862 (1.532-9.735)	**0.008**	3.806 (1.427-10.151)
**Vaccination**	**0.002**	0.329 (0.162-0.665)	**<0.001**	0.293 (0.141-0.607)
**3 doses**	0.216	0.503 (0.170-1.495)	0.072	0.342 (0.106-1.102)
**4 doses**	0.750	1.207 (0.379-3.841)	0.862	1.109 (0.345-3.566)
**Antiviral home**	0.070	0.152 (0.020-1.164)	0.059	0.139 (0.018-1.075)
**mAbs home**	0.059	0.140 (0.018-1.075)	0.053	0.133 (0.017-1.024)
**Any home treatment**	**<0.001**	0.143 (0.049-0.415)	**<0.001**	0.130 (0.044-0.381)

The bold values refers to statistical significant data when the corresponding p value is < 0.05.

When considering the whole cohort and last available data before SARS-CoV-2 infection, we did not find significant differences in absolute lymphocyte counts, percentage of CD3+, CD4+ and CD8+ lymphocytes, switched memory B cells, IgM memory B cells, CD21lo B-cells, serum IgG-TL and IgA between hospitalized and non-hospitalized patients. Of note, B-cell percentage tended to be lower in hospitalized patients [3.5% (1.0-9.0) versus 7% (3–12); p=0.051]. Data are recapitulated in **Table 5**.

**Table 5:** Mann-Whitney U test for comparison of immunologic parameters between hospitalized and non-hospitalized patients in the whole CVID cohorts.

Whole cohort	Hospitalized	Non hospitalized	p value
**Duration (days)**	28 (20-42.5)	14 (10.0-22.0)	**<0.001**
**Lymphocyte count (pre-infection)**	1305 (837.5-2070)	1465 (1150-2017.5)	0.299
**CD3%**	80 (75.0-84.0)	77 (70.0-82.0)	0.111
**CD4%**	39.5 (30.2-56.0)	42 (35.0-49.0)	0.754
**CD8%**	32.5 (19.0-45.7)	31 (22.25-36.0)	0.732
**B cell %**	3.5 (1.0-9.0)	7 (3.0-12.0)	**0.051**
**SmB %**	6.0 (2.0-10.0)	3.0 (1.0-10.0)	0.522
**IgM memory B %**	6.5 (0.25-14.2)	7.0 (2.0-17.9)	0.383
**CD21lo B %**	10 (1.5-20.0)	7.0 (3.0-15.0)	0.661
**IgG-TL (mg/dl)**	800 (600-1000)	741 (570-890)	0.276
**IgA (mg/dl)**	6.0 (1.0-21.0)	8.0 (1.0-30.0)	0.524

Of note, superinfections (OR 33.378, 95%CI 7.499-148.572; p<0.001), ESLD (OR 14.173, 95%CI 2.211-90.869; p=0.005) and chronic immunosuppressive treatment (OR 5.894, 95%CI 1.441-24.114; p=0.014) were also found associated with an increased risk of severe disease, when adjusted for sex and age. Superinfections retained statistical significance also when adjusted for sex, age, ESLD and chronic immunosuppressive treatment (OR 23.163, 95%CI 4.592-116.832; p<0.001).

Analyzing the impact of comorbidities and clinical features on mortality, Fisher exact t test showed an increased mortality in patients with chronic lung disease (p=0.013), bronchiectasis (p=0.008), previous cardiovascular events (p=0.043) and superinfections (p=0.003) (**Supplementary Table 9**).

We then performed univariate logistic regression analysis suggesting an association between age (OR 1.077, 95%CI 1.007-1.152; p=0.031), ESLD (OR 11.792, 95%CI 1.141-121.89; p=0.038), previous CV events (OR 9.439, 95%CI 1.486-59.950; p=0.017) and superinfections (OR 22.417, 95%CI 3.519-142.813; p<0.001), and an increased risk of mortality (**Table 6** and **Figure 2**). However, when these analyses were adjusted, respectively for sex and for sex and age, only age and superinfections retained significance.

**Table 6:** Univariate logistic regression analysis for mortality in the whole CVID cohort **(**waves 1-4).

Whole cohort	Unadjusted		Adjusted for age and sex	
	p value	OR (IC95%)	p value	OR IC(95%)
**Age**	**0.031**	1.077 (1.007-1.152)	**0.019**	1.092* (1.014-1.175)
**Sex (F)**	0.462	1.968 (0.324-11.957)	0.185	0.272** (0.040-1.862)
**Chronic lung disease**	0.995	67.9x10^6^	0.995	44.2x10^6^
**Bronchiectasis**	0.995	75.5x10^6^	0.995	46.9x10^6^
**GLILD**	0.788	1.356 (0.148-12.410)	0.675	1.632 (0.166-16.085)
**ESLD**	**0.038**	11.792 (1.141-121.891)	0.127	7.146 (0.572-89.335)
**Complicated phenotype**	0.930	1.085 (0.178-6.595)	0.993	1.008 (0.160-6.351)
**Chronic Immunosuppressive treatment**	**0.059**	6.811 (0.931-49.830)	**0.055**	7.636 (0.955-61.040)
**IgG-TL**	0.208	1.002 (0.999-1.005)	0.128	1.003 (0.999-1.007)
**Age>65**	0.260	2.386 (0.463-17.386)	0.181	3.616* (0.549-23.803)
**Obesity**	0.579	0.533 (0.058-4.912)	0.629	0.571 (0.058-5.573)
**Arterial hypertension**	0.079	5.068 (0.829-30.974)	0.576	1.826 (0.221-15.067)
**Diabetes**	0.998	0.000	0.998	0.000
**Previous CV events**	**0.017**	9.439 (1.486-59.950)	0.297	3.165 (0.363-27.619)
**Atherosclerosis**	0.376	2.740 (0.294-25.498)	0.861	0.803 (0.068-9.483)
**Superinfections**	**<0.001**	22.417 (3.519-142.813)	**0.003**	20.120 (2.832-142.919)

The bold values refers to statistical significant data when the corresponding p value is < 0.05.

#### Reinfection

During the whole study period, 44 re-infections were registered in 42 patients (14.3%), with more cases documented in IT-C than in the NL-C (38/218 vs 4/76; p=0.008). All but one case of re-infections occurred during the Omicron wave. The disease was mild in 41 (93.2%) and moderate in 3 (6.8%) cases. No severe disease or death were recorded. The median time of RT-PCR positivity was 10 days (range 8-16). Superinfections were recorded in 5 cases (11.9%). Interesting to note, only one patient was vaccinated with 4 doses at re-infection. The re-infection rate in the IT-C (17.4%) was significantly higher compared to that reported in the Italian population at the same date (5.9%; p<0.001).

## Discussion

At the beginning of the COVID-19 pandemic, all countries implemented different policies to limit SARS-CoV-2 spreading. Since January 2021 the pandemic course has been influenced by the introduction of vaccination (35–37), and by the emergence of new SARS-CoV-2 strains with increased infectivity, different severity and potential ability to escape host immunity (38). In particular, the Omicron variant has been associated with lower severity (17).

At first, patients with IEIs were particularly monitored for the *a priori* risk of severe disease after SARS-CoV-2 infection (39, 40). During the primary phase of virus spreading (Wuhan and Alpha waves) lifestyle of patients changed completely: social distancing, home isolation and PPE were strictly respected. These measures probably contributed to the lower incidence of SARS-CoV-2 infection in IEI patients when compared to the general population during the first waves of COVID-19. Over the last year, the reduction of containment measures and the spreading of the Omicron lineage (and its sub-variants) resulted in an exponential growth of confirmed and, likely, of unofficial COVID-19 cases in these patients.

In literature, various national studies described the severity of COVID-19 in immunocompromised patients and the impact of different comorbidities, specific treatments and vaccination status, showing variable outcomes (9, 15, 27, 41, 42). To our knowledge, this is the first European multicentric real-life study, comparing the course of SARS-CoV-2 infection in a cohort of CVID patients enrolled in two different countries who adopted, at least in part, different policies of COVID-19 pandemic management.

In previous studies, the immunogenicity of mRNA vaccines in patients with CVID has already been demonstrated (20, 21, 41, 43). Moreover, vaccinated patients with IEIs have been shown to present a less severe disease course, also when compared with pre-immunization studies in unvaccinated patients (42, 44). Since the beginning of the SARS-CoV-2 pandemic, different specific therapies have also become available for vulnerable patients. In particular, the use of antiviral therapy has been shown to reduce the risk of severe SARS-CoV-2 disease and shorten RT-PCR positivity, while mAbs administration reduced severity and risk of hospitalization in IEIs patients (27).

In our study, the proportion of CVID patients infected by SARS-CoV-2 was similar to the general population in both national sub-cohorts. As expected, the vast majority of cases were recorded during the Omicron wave. The difference in epidemiologic impact of the Omicron strain between the two national sub-cohorts may be related to a different surveillance strategy started from Spring 2022 in the Netherlands, with a shift towards self-testing limited to symptomatic patients. This could also explain the different re-infection rates, since re-infections occurred almost exclusively during the Omicron wave. The demographic characteristics, cardiovascular and pulmonary co-morbidities of the two sub cohorts were comparable, except for older age and higher prevalence of “complicated” clinical phenotype in the IT-C. The vaccination adherence was similar between the sub-cohorts and higher than the general population. This confirms the success of immunization campaigns in vulnerable patients.

With regard to antiviral drugs and mAbs, management policies differed between the two countries. In the Netherlands, specific treatments were reserved to the hospitalized patients, whereas in Italy these therapies became widely available for CVID patients in extra-hospital settings, particularly during the Omicron wave. As a consequence, we found a significantly higher usage of both antiviral and mAbs treatments in the IT-C cohort. The enrolled patients presented a mild disease course in the vast majority of cases. However, hospital and ICU admission occurred in a higher percentage of cases as compared to the general population from the two countries. These data confirm that patients with CVID are at greater risk of a worse disease course (7–9). In line with that, during the study period, mortality rate was also higher than in the general population, despite being lower than that reported in other CVID cohorts (8).

Of note, despite similar baseline characteristics and a different use of SARS-CoV-2 specific treatments, the clinical outcome of the infection was not different between the two sub-cohorts, both considering the whole period and the specific waves. We thus decided to investigate whether specific risk factors or treatments might have impacted on the risk of hospitalization, complications and mortality.

Starting from hospitalization, as demonstrated in previous studies, we found that chronic lung disease, “complicated” phenotype and chronic immunosuppressive treatment impacted on this outcome, as well as cardiovascular comorbidities, in particular atherosclerotic disease, arterial hypertension and previous cardiovascular events (3, 13, 16). Chronic lung disease and immunosuppressive treatment, in particular, appeared to be the strongest predictors. Moreover, the hospitalized patients tended to have lower B cell percentages, as previously suggested by Milota et al, and longer RT-PCR positivity (9); of note, serum IgA and IgG-TL were not found significantly different. Considering the above-mentioned risk factors, only the prevalence of “complicated” phenotype was different between IT-C and NL-C. We then showed that vaccination reduces the risk of hospitalization when considering the whole cohort, but we could not confirm the protective role during the omicron wave. This is likely due to the almost complete vaccination coverage at that time and to the contemporary reduction of virulence of the said strain. Home based mAbs treatment was effective when considering patients infected after the spreading of Delta variant but neither mAbs, nor antivirals were shown to impact on the risk of hospitalization during the Omicron wave. The loss of efficacy of mAbs during the Omicron wave has been largely reported against the more recent sub variants (45, 46). However, when considered together, the two home treatment strategies reduced the risk of hospital admission also during the Omicron period.

Moving to mortality as an outcome, the risk factors were older age, pre-existing chronic lung disease, and bacterial superinfections. Interestingly, older age was not found as a risk factor for hospitalization. However, hospitalized patients were more frequently complicated in terms of clinical phenotype and hospitalization was a significant risk factor for superinfections. This might explain why older hospitalized patients were at higher risk for mortality, also taking into account a likely age-related higher frequency of non CVID-related comorbidities (e.g. cardiovascular). Considering the low numbers and the date of occurrence of the events, it has not been possible to assess the impact of vaccination and specific treatments on this outcome. Bacterial superinfections have already been reported as impacting on the outcome of hospitalized patients, especially if immunocompromised during viral infections including SARS-CoV-2 (47).

Since superinfections were the most common reported complications of COVID-19 in our cohort, apart from acute respiratory failure, and impacted on the risk of severe disease and mortality, we then explored their major determinants. Of note, despite different IgG-TL in the two sub cohorts, we did not observe a different rate of superinfection. Moreover, IgG-TL was not lower in superinfected patients, while a lower B cell percentage was found. Superinfections were more common in hospitalized patients, those with chronic lung disease, “complicated” phenotype, chronic immunosuppressive treatment and previous CV events. A longer duration of SARS-CoV-2 swab positivity was also associated with this complication. Noteworthy, despite a progressive reduction of the RT-PCR positivity due to the change of viral strains over time, we showed that vaccination with at least 3 doses shortened the time of RT-PCR positivity during the Delta and Omicron waves. Interestingly, this effect was enhanced by antiviral treatment but not by mAbs.

All these considered, the two sub-cohorts had similar COVID-19 outcomes despite a clearly different treatment approach. This does not prove that the use of antivirals and mAbs is of no relevance in CVID patients. It is known that the efficacy of mAbs is closely dependent on their use on the appropriate VOC, as we previously demonstrated the impact of Sotrovimab against Omicron BA.1 (27).

As far as antivirals are concerned and since superinfection is correlated with mortality, we can speculate that shortening time of RT-PCR positivity could be a valuable result in CVID patients. Of note, IT-C patients were older and more frequently “complicated” but did not have higher hospitalization, superinfection or mortality rates. This could have been partly due to the home-based treatment, as suggested by the analysis of the impact of home treatment on the risk of hospitalization in the IT-C. Interestingly, we observed that these treatments were given preferentially to patients with a “complicated” phenotype, possibly implicating a different approach to this specific subgroup of CVID patients in clinical practice. This supports the hypothesis that, rather than being used indiscriminately on the basis of a CVID diagnosis, these therapies could be of additional benefit in specific patients’ subgroups according to pre-existing conditions and treatments, which is in line with policy in the general population. Apart from this, also the current variant should be taken into account when considering specific treatments, as the effect may be different for different variants. Another point to be considered is that a complicated clinical phenotype is known to impact the response to vaccination in CVID patients, also due to the concomitant use of immunosuppressive medications (21). ​​Cellular response to SARS-CoV-2 is probably one of the key determinants of severe disease protection, especially months after viral and/or vaccine exposure in a context of waning or absent humoral immunity. In addition to antibodies and memory B cells, memory T cells can contribute to protection upon SARS-CoV-2 exposure, and the latter has also been shown to be less affected by VOCs ability to overcome the protective effect of neutralizing antibodies produced as a result of natural infection and/or vaccination (48). Whether the degree of T or B cell response to vaccination impacts on disease severity in CVID patients is still unknown and deserves further studies, but we may also hypothesize that the specific treatment could be of help in case of poor response to vaccination (24).

Based on our findings we suggest a tailored therapeutic approach, providing early administration of home therapy to those patients with known risk factors for severe disease, including complicated phenotype, as well as non-vaccinated or non-responders to vaccination. Pre-exposure prophylaxis could be another reasonable approach in these patients, but the increasing titres of polyclonal anti-SARS-CoV-2 antibodies in the preparations of IgRT may also be of clinical significance in this view (49, 50).

We are aware that our study presents some limitations. First of all, the different rules in the two countries could have affected the number of detected cases and have clearly influenced the treatment approach. Genotype assessment of SARS-CoV-2 variant was not systematically conducted, so that the classification of SARS-CoV-2 strain has been performed mainly based on epidemiology. A selection bias may have occurred since not all patients in the NL-C have given their consent to publish data. We also did not systematically evaluate vaccination responses in our patients, which could have influenced the outcome of vaccination considered. Finally, the reduction in mortality over the study period might also have been influenced by a harvesting effect, as well by the characteristics of the Omicron strain.

However, the comparison of two national cohorts, taking advantage of the different adopted approaches, may strengthen the quality of the results. In conclusion, we confirmed that CVID patients have a higher risk of severe outcomes during COVID-19 course, related to specific risk factors, than the general population. Disease severity is reducing over time, due to the new variants and to specific interventions. Vaccination coverage is higher than 90% in CVID patients and shortens RT-PCR positivity. Specific outpatient treatment can impact on the risk of hospitalization with antivirals significantly reducing swab positivity duration also in vaccinated patients. Pre-existing CVID-related and cardiovascular co-morbidities increase the risk of severe COVID-19 and its complications, in particular superinfections. Our results point out that more emphasis about home treatment should probably be reserved to those at-risk patients.

## Data availability statement

The raw data supporting the conclusions of this article will be made available by the authors, without undue reservation and upon reasonable request.

## Ethics statement

The studies involving human participants were reviewed and approved by Ethical Committee of the Sapienza University of Rome, Italy; Comitato Etico delle Province di Treviso e Belluno, Italy (793/CE Marca Trevigiana); Erasmus University MC Rotterdam (METC-2013-026). The patients/participants provided their written informed consent to participate in this study.

## Author contributions

CM, DF, FC, IQ, GS, VD conceptualized the study. VD, SG, MR, RS designed the study protocol. PB, AV, GL, AP, GC, LL, BP, CD, Gd’I, recruited patients and collected data. CM, DF, VD and FC performed the statistical analysis. AV, CM, PB, DF, VD, GC, RS,VD, FC prepared the first draft of the manuscript. All authors reviewed the manuscript before publication. All authors contributed to the article and approved the submitted version.
