# Supplementary material for: A beacon in the dark: COVID-19 course in CVID patients from two European countries: Different approaches, similar outcomes
Source: Front Immunol. 2023 Feb 8;14:1093385. doi: 10.3389/fimmu.2023.1093385 (PMC9944020; doi:10.3389/fimmu.2023.1093385)
Supplement: Supplementary file 1 [file DataSheet_1.docx]

**SUPPLEMENTARY TABLES**

**SUPPLEMENTARY TABLE 1**: infection and mortality rate in CVID and general population from March 1st, 2020 to September 1st, 2022. *Vaccination: at least 2 doses.*

|  | **Whole cohort (N=773)** | **IT-C**  **(N=497)** | **NL-C**  **(N=276)** |
| --- | --- | --- | --- |
| **CVID infection rate, n (%)** | 329 (42.5) | 218 (43.9) | 111 (40.2) |
| **CVID hospitalization rate, n (%)** | 37 (12.6) | 25 (11.5) | 12 (15.78) |
| **CVID mortality rate, n (%)** | 5 (1.5) | 4 (1.8) | 1 (0.9) |
| **National infection rate, n (%)** | - | 22 003 228 (37.3) | 8386061 (48.7) |
| **National hospitalization rate** | - | 1.5% at October 1st 2022 | N/A |
| **National mortality rate, n (%)** | - | 171895 (0.78) | 22605 (0.27) |
| **CVID vaccination rate (2 doses) at infection (only for omicron wave)** | 191/204 (93.6) | 150/159 (94.3) | 41/45 (91.1) |
| **National vaccination rate (2 doses) at September 1st, 2022** | - | 49966159 (84.7) | 11 957 652 (68.5) |

**SUPPLEMENTARY TABLE 2** non CVID-related risk factors for severe COVID-19 course

|  | **Whole cohort (N=294)** | **IT-C**  **(N=218)** | **NL-C**  **(N=76)** | **p value** |
| --- | --- | --- | --- | --- |
| Aged>65 (%) | 57 (19.4) | 46 (21) | 11 (14.4) | 0.241 |
| Obesity (%) | 35 (11.9) | 24 (11) | 11 (14.5) | 0.416 |
| Arterial hypertension (%) | 69 (23.4) | 55 (25.2) | 14 (20) | 0.272 |
| Diabetes (%) | 21 (7.1) | 14 (6.4) | 7 (9.2) | 0.441 |
| Previous CV events (%) | 21 (7.1) | 15 (6.9) | 6 (7.9) | 0.798 |
| Atherosclerotic disease (%) | 25 (8.5) | 18 (8.2) | 7 (9.3) | 0.812 |

**SUPPLEMENTARY TABLE 3:** Narrative review of the 5 dead patients.

| **Cohort** | **AGE** | **SEX** | **Lung comorbidities** | **Cardiovascular Comorbidity** | **Clinical phenotype** | **Wave** | **Vaccination Doses at infection** | **COVID-19 complications** |
| --- | --- | --- | --- | --- | --- | --- | --- | --- |
| **IT-C** | 52 | M | Bronchiectasis, GLILD, ESLD, Previous Lung Transplant | None | Complicated  (LP) | 1-2 | 0 | RF, SI |
| **IT-C** | 59 | F | Bronchiectasis | HTN, previous CV event | Infection only | 1-2 | 0 | ACS |
| **NL-C** | 91 | F | Bronchiectasis | HTN | Infection only | 3 | 3 | RF |
| **IT-C** | 73 | M | Bronchiectasis | HTN, previous CV event | Infection only | 3 | 2 | RF, SI |
| **IT-C** | 50 | M | Bronchiectasis  GLILD | None | Complicated  (LP); cancer | 4 | 3 | RF, SI |

HTN=arterial hypertension; CV: cardiovascular; LP= lymphoproliferation; RF= respiratory failure; SI=superinfection; ACS= acute coronary syndrome.

**SUPPLEMENTARY TABLE 4A-D:** Comparison between IT-C and NL-C during different SARS-CoV-2 waves. *ICU: intensive care unit*

| **SUPPLEMENTARY TABLE 4A** | | **Whole cohort** | **IT-C** | **NL-C** | **p value** |
| --- | --- | --- | --- | --- | --- |
| **Wuhan/Alpha**  **(wave 1 and 2)** | **Number of patients** | 65 | 47 | 18 |  |
|  | **Mild** | 46 | 32 | 14 | 0.550 |
|  | **Moderate** | 12 | 10 | 2 | 0.485 |
|  | **Severe** | 6 | 4 | 2 | 0.666 |
|  | **Hospitalized** | 18 | 14 | 4 | 0.758 |
|  | **ICU** | 3 | 3 | 0 | 0.555 |
|  | **Dead** | 2 | 2 | 0 | 0.520 |
|  | **mAbs** | 9 | 9 | 0 | 0.053 |
|  | **mAbs home** | 7 | 7 | 0 | 0.176 |
|  | **Antiviral** | 9 | 8 | 1 | 0.425 |
|  | **Antiviral home** | - | - | - | - |

| **SUPPLEMENTARY TABLE 4B** | | **Whole cohort** | **IT-C** | **NL-C** | **p value** |
| --- | --- | --- | --- | --- | --- |
| **Delta**  **(wave 3)** | **Number of patients** | **26** | **13** | **13** |  |
|  | **Mild** | 20 | 10 | 10 | 1.000 |
|  | **Moderate** | 3 | 1 | 2 | 1.000 |
|  | **Severe** | 3 | 2 | 1 | 1.000 |
|  | **Hospitalized** | 6 | 3 | 3* | 1.000 |
|  | **ICU** | 2 | 2 | 0 | 0.480 |
|  | **Dead** | 2 | 1 | 1 | 1.000 |
|  | **mAbs** | 12 | 7 | 5* | 0.695 |
|  | **mAbs home** | 6 | 6 | 0 | **0.015** |
|  | **Antiviral** | 1 | 0 | 1 | 1.000 |
|  | **Antiviral home** | 0 | 0 | 0 | - |

* 2 patients were admitted with mild disease and received mAbs treatment and were discharged right after the treatment; for this reason, they have not been considered as hospitalized when using hospitalization as clinical outcome

| **SUPPLEMENTARY TABLE 4C** | | **Whole cohort** | **IT-C** | **NL-C** | **p value** |
| --- | --- | --- | --- | --- | --- |
| **Delta + Omicron**  **(wave 3 and 4)** | **Number of patients** | **229** | **171** | **58** |  |
|  | **Mild** | 210 | 160 | 50 | 0.098 |
|  | **Moderate** | 16 | 9 | 7 | 0.131 |
|  | **Severe** | 3 | 2 | 1 | 1.000 |
|  | **Hospitalized** | 19 | 11 | 8 | 0.098 |
|  | **ICU** | 4 | 4 | 0 | 0.575 |
|  | **Dead** | 3 | 2 | 1 | 1.000 |
|  | **mAbs** | 73 | 66 | 7 | **<0.001** |
|  | **mAbs home** | 60 | 60 | 0 | **<0.001** |
|  | **Antiviral** | 63 | 62 | 1 | **<0.001** |
|  | **Antiviral home** | 57 | 57 | 0 | **<0.001** |

| **SUPPLEMENTARY TABLE 4D** | | **Whole cohort** | **IT-C** | **NL-C** | **P (chi/fisher)** |
| --- | --- | --- | --- | --- | --- |
| **Omicron**  **(wave 4)** | **Number of patients** | **204** | **159** | **45** |  |
|  | **Mild** | 191 | 151 | 40 | 0.166 |
|  | **Moderate** | 13 | 5 | 8 | 0.166 |
|  | **Severe** | 0 | 0 | 0 | - |
|  | **Hospitalized** | 13 | 8 | 5 | 0.166 |
|  | **ICU** | 2 | 0 | 0 | 1.000 |
|  | **Dead** | 1 | 1 | 0 | 1.000 |
|  | **mAbs** | 62 | 60 | 2 | **<0.001** |
|  | **mAbs home** | 55 | 55 | O | **<0.001** |
|  | **Antiviral** | 62 | 62 | 0 | **<0.001** |
|  | **Antiviral home** | 57 | 57 | 0 | **<0.001** |

**SUPPLEMENTARY TABLE 5**: Univariate logistic regression analysis for hospitalization in CVID cohort from Delta period (considering waves 3 and 4).

| **Delta + Omicron** | **Unadjusted** | | **Adjusted for age and sex** | |
| --- | --- | --- | --- | --- |
|  | **p value** | **OR (IC95%)** | **p value** | **OR IC(95%)** |
| **Age** | 0.088 | 1.027  (0.996-1.059) | 0.078 | 1.029*  (0.997-1.061) |
| **Sex (F)** | 0.858 | 0.917  (0.354-2.374) | 0.615 | 0.778**  (0.292-2.070) |
| **Chronic lung disease** | 0.069 | 2.424  (0.935-6.289) | 0.116 | 2.179  (0.826-5.449) |
| **Bronchiectasis** | **0.024** | 3.000  (1.153-7.805) | **0.046** | 2.688  (1.018-7.098) |
| **GLILD** | **0.005** | 4.317  (1.554-11.968) | **0.004** | 4.742  (1.661-13.535) |
| **ESLD** | **0.002** | 39.187  (3.853-398.589) | **0.004** | 35.258  (3.194-389.210) |
| **Complicated phenotype** | **0.008** | 3.900 (1.424-10.683) | **0.011** | 3.737  (1.356-10.298) |
| **Chronic immunosuppressive treatment** | **<0.001** | 8.222  (3.047-22.187) | **<0.001** | 8.400  (3.015-23.403) |
| **IgG-TL** | 0.724 | 1.000  (0.998-1.002) | 0.527 | 1.001  (0.999-1.003) |
| **Age>65** | 0.264 | 1.792  (0.644-4.990) | 0.243 | 1.863*  (0.655-5.297) |
| **Obesity** | 0.858 | 0.871  (0.190-3.995) | 0.817 | 0.835  (0.181-3.858) |
| **Arterial Hypertension** | **0.041** | 2.744  (1.041-7.234) | 0.205 | 2.091  (0.669-6.537) |
| **Diabetes** | 0.654 | 1.426  (0.302-6.730) | 0.992 | 0.992  (0.200-4.920) |
| **Previous CV events** | **<0.001** | 16.162  (5.224-49.999) | **<0.001** | 16.091  (4.505-57,475) |
| **Atherosclerotic disease** | **<0.001** | 8.083  (2.749-23.766) | **0.001** | 6.875  (2.175-21.73) |
| **Vaccination**  **(at least 2 doses)** | 0.663 | 1.585 (0.199-12.613) | 0.936 | 1.091  (0.130-9.157) |
| **Antiviral home** | 0.070 | 0.152  (0.020-1.164) | 0.059 | 0.139  (0.018-1.075) |
| **mAbs home** | 0.059 | 0.140  (0.018-1.075) | 0.053 | 0.133  (0.017-1.024) |
| **Any home treatment** | **0.003** | 0.101 (0.023-0.448) | **0.002** | 0.092 (0.021-0.412) |
| **3 doses** | 0.216 | 0.503  (0.170-1.495) | 0.072 | 0.342  (0.106-1.102) |
| **4 doses** | 0.750 | 1.207  (0.379-3.841) | 0.862 | 1.109  (0.345-3.566) |

*adjusted for sex; **adjusted for age. *GLILD: Granulomatous and Lymphocytic Interstitial Lung Disease; ESLD: End-Stage-Lung Disease, IgG-TL: IgG Trough level; CV: cardiovascular; mAbs: monoclonal antibodies.*

**SUPPLEMENTARY TABLE 6:** Univariate logistic regression analysis for hospitalization in CVID cohort during Omicron period (wave 4).

| **Omicron** | **Unadjusted** | | **Adjusted for age and sex** | |
| --- | --- | --- | --- | --- |
|  | **p value** | **OR (IC95%)** | **p value** | **OR IC(95%)** |
| **Age** | 0.053 | 1.039 (0.9999-1.081) | 0.052 | 1.040*  (1.000-1.043) |
| **Sex (F)** | 0.954 | 0.967 (0.305-3.067) | 0.761 | 0.831**  (0.253-2.732) |
| **Chronic lung disease** | 0.078 | 2.829  (0.891-8.986) | 0.129 | 2.488  (0.768-8.062) |
| **Bronchiectasis** | 0.115 | 2.486 (0.802-7.713) | 0.230 | 2.034 (0.638-6.492) |
| **GLILD** | **0.004** | 6.007 (1.7777-20.311) | **0.004** | 6.484 (1.822-23.074) |
| **ESLD** | **0.005** | 34.545 (2.904-410.957) | **0.024** | 19.626 (1.478-260,630) |
| **Complicated phenotype** | **0.005** | 6.772  (1.800-25.479) | **0.008** | 6,067 (1.595-23.80) |
| **Chronic immunosuppressive treatment** | **0.001** | 6.939  (2.130-22.602) | **0.003** | 6.709  (1.952-23.059) |
| **IgG-TL** | 0.179 | 0.998  (0.996-1.001) | 0.268 | 0.999  (0.996-1.001) |
| **Age>65** | 0.467 | 1.577 (0.462-5.376) | 0.467 | 1.590*  (0.466-5.543) |
| **Obesity** | 0.641 | 0.609 (0.076-4.902) | 0.625 | 0.594 (0.073-4.838) |
| **Arterial Hypertension** | 0.182 | 2.217 (0.689-7.134) | 0.756 | 1.241 (0.317-4.855) |
| **Diabetes** | 0.352 | 2.133 (0.432-10.526) | 0.705 | 1.377 (0.263-7.205) |
| **Previous CV events** | **<0.00**1 | 12.569 (3.417-46.237) | **0.004** | 10.481 (2.116-51.904) |
| **Atherosclerotic disease** | **0.001** | 7.812  (2.254-27.076) | **0.014** | 5.728  (1.422-23.080) |
| **Vaccination** | 0.999 | N/A | 0.999 | N/A |
| **3 doses** | 0.998 | N/A | 0.998 | N/A |
| **4 doses** | 0.354 | 1.789  (0.523-6.123) | 0.437 | 1.638  (0.472-5.685) |
| **mAbs home** | 0.136 | 0.208  (0.026-1.642) | 0.119 | 0.192  (0.024-1.526) |
| **Antiviral home** | 0.126 | 0.199 (0.025-1.570) | 0.106 | 0.179  (0.022-1.437) |
| **Any home treatment** | **0.012** | 0.140 (0.030-0.648) | **0.008** | 0.123 (0.026-0.581) |

*adjusted for sex; **adjusted for age. *GLILD: Granulomatous and Lymphocytic Interstitial Lung Disease; ESLD: End-Stage-Lung Disease, IgG-TL: IgG Trough level; CV: cardiovascular; mAbs: monoclonal antibodies.*

**SUPPLEMENTARY TABLE 7:** duration of Sars-CoV-2 RT-PCR positivity with different variants.

|  | **Median duration (days)** | **Max duration (days)** |
| --- | --- | --- |
| **Wuhan-Alpha** | 22.0 (16.0-30.5) | 69 |
| **Delta** | 17.0 (13.5-20.0) | 42 |
| **Omicron** | 14.0 (9.8-22.0) | 84 |

**SUPPLEMENTARY TABLE 8**. Fisher’s exact test for superinfections in the whole cohort

|  | **p value** |
| --- | --- |
| **Age>65** | 0.236 |
| **Sex** | 0.820 |
| **Chronic lung disease** | **0.005** |
| **Bronchiectasis** | **<0.001** |
| **GLILD** | **0.002** |
| **ESLD** | **<0.001** |
| **Complicated phenotype** | **0.019** |
| **Chronic immunosuppressive treatment** | **<0.001** |
| **Hospitalization** | **<0.001** |
| **Previous CV events** | **0.011** |

**SUPPLEMENTARY TABLE 9:** Fisher’s exact test for mortality in the whole CVID cohort.

|  | **p value** |
| --- | --- |
| **Age>65** | 0.249 |
| **Sex** | 0.656 |
| **Chronic lung disease** | **0.013** |
| **Bronchiectasis** | **0.008** |
| **GLILD** | 0.576 |
| **ESLD** | 0.114 |
| **Chronic immunosuppressive treatment** | 0.087 |
| **Arterial Hypertension** | 0.086 |
| **Previous CV events** | **0.043** |
| **Atherosclerotic disease** | 0.363 |
| **Obesity** | 0.472 |
| **Diabetes** | 1.000 |
| **Superinfections** | **0.003** |
| **Home treatment** | 0.079 |
